# Eribulin as a first-line treatment for soft tissue sarcoma patients with contraindications for doxorubicin

**DOI:** 10.1038/s41598-020-77898-y

**Published:** 2020-12-01

**Authors:** Kenji Tsuchihashi, Hitoshi Kusaba, Tomoyasu Yoshihiro, Toshifumi Fujiwara, Nokitaka Setsu, Makoto Endo, Yoshihiro Matsumoto, Takashi Imajima, Yudai Shinohara, Mamoru Ito, Satoru Yamaga, Kenro Tanoue, Kohei Arimizu, Hirofumi Ohmura, Fumiyasu Hanamura, Kyoko Yamaguchi, Taichi Isobe, Hiroshi Ariyama, Yasuharu Nakashima, Koichi Akashi, Eishi Baba

**Affiliations:** 1 Department of Hematology, Oncology and Cardiovascular Medicine, Kyushu University Hospital, Fukuoka, 812-8582 Japan; 2 Department of Medicine and Biosystemic Science, Graduate School of Medical Sciences, Kyushu University, Fukuoka, 812-8582 Japan; 3 Department of Orthopaedic Surgery, Graduate School of Medical Sciences, Kyushu University, Fukuoka, 812-8582 Japan; 4 Department of Oncology and Social Medicine, Graduate School of Medical Sciences, Kyushu University, Fukuoka, 812-8582 Japan

**Keywords:** Sarcoma, Chemotherapy

## Abstract

Doxorubicin is a first-line therapy for patients with unresectable advanced soft tissue sarcoma (STS). However, because of cardiotoxicities, it is not used for patients with cardiac problems. Eribulin has exhibited efficacy for advanced STS in second- or later-line treatments. In the present study, we retrospectively analyzed the efficacy and safety of first-line eribulin therapy for patients with advanced STS unable to receive doxorubicin. Six of 28 patients who received eribulin as any line treatment received eribulin as a first-line treatment. The reasons for avoiding doxorubicin were as follows: cardiac problems for four patients and advanced age for two. Median progression-free survival (PFS) of the patients who received eribulin as first-line and, second or later-line therapy were 9.7 months (95% CI: 1.0-not reached) and 3.9 months (95% CI: 2.7–5.9), which were not significantly different. The reasons for discontinuation of eribulin were disease progression and adverse events (2 fatigue and 1 neuropathy) for three patients each. No treatment-related cardiotoxicity was observed. The findings of this study indicated that eribulin exhibits meaningful efficacy for the patients with contraindications for doxorubicin as a first-line treatment without cardiac adverse events. However, appropriate safety management is necessary because older patients are typically among those intolerable of doxorubicin.

## Introduction

Soft tissue sarcomas (STS) are heterogenous tumors with over 50 subtypes^1^. Doxorubicin is globally applied as a first-line therapy for patients with unresectable advanced STS. However, doxorubicin has a side effect of cardiotoxicity and is generally contraindicated for patients with current or previous abnormal cardiac function^2^. Clinical trials on doxorubicin have excluded patients with abnormal left ventricular or cardiac ejection fraction^3,4^. STS is common in older patients, and the proportion of patients with STS aged > 60 years exceeds 50% in Japan^5,6^. Older patients have an increased risk of doxorubicin-induced heart failure^7^, and old age is reported to be associated with increased hematological toxicity by anthracyclines^8^. Accordingly, the use of doxorubicin tends to be avoided in older populations. Thus, the treatment strategy for the patients with STS with cardiac comorbidities or the aged remains unclear, and is therefore an important issue.

Other chemotherapies for advanced STS are histology-driven, and the application of each drug differs slightly between countries. In Japan, eribulin, pazopanib, trabectedin and ifosfamide are applied^1,9–12^. Eribulin is a microtubule inhibitor that inhibits the growth of microtubules, causing G2/M cell cycle arrest. Eribulin significantly prolonged overall survival (OS) in previously treated patients with advanced liposarcoma (L-sarcoma) or leiomyosarcoma (LMS) compared with dacarbazine in a phase III trial. A phase II trial in Japan also showed the efficacy of eribulin for patients with advanced STS^9,12^. In Japan, eribulin is approved for any kinds of soft tissue sarcoma. On the other hand, for example, eribulin is approved only for liposarcoma in the U.S. based on the data preplanned, exploratory subgroup analyses of OS. Eribulin has been also applied for patients with metastatic breast cancer and its safety and efficacy have been confirmed for older patients^13^.

In the present study, we retrospectively investigated the safety and efficacy of first-line eribulin in patients with STS who were unable to receive doxorubicin because of cardiac comorbidities or advanced age.

## Patients and methods

### Patients

We collected data from 28 patients who started eribulin treatment between April 2016 and December 2018 at our institution. The cutoff date was November 2019. Six of 28 patients who received eribulin as any line treatment received eribulin as a first-line treatment. The eligibility criteria were age ≥ 20 years, histologically proven metastatic or recurrent STS, and receiving eribulin and having sufficient organ function tolerable to chemotherapy.

### Treatments

All patients received 1.4 mg/m^2^ eribulin as a 5-min intravenous infusion on days 1 and 8 every 3 weeks. Initial dose reduction was allowed according to patient status at the discretion of the investigators. Outpatients generally visited the investigators on days 1 and 8 every 3 weeks. At each visit, physical examinations, laboratory tests, and assessments for adverse events (AEs) were performed. The treatment was continued until disease progression, unacceptable toxicity, or a decision to discontinue by the patient or investigator. Dose reduction and treatment delay were performed according to the manufacturer’s instructions.

### Assessment

Medical information of each patient was retrospectively obtained by electronic records. Tumor lesions were assessed by computed tomography (CT) every 2–3 months. In cases of worsening symptoms or laboratory findings, CT was performed. Progression-free survival (PFS) and OS were defined as the period from the initiation of eribulin to the day of tumor progression or the day of death from any cause, respectively. Tumor responses were assessed using the Response Evaluation Criteria in Solid Tumors (RECIST) version 1.1. Adverse events during the therapy were evaluated according to the National Cancer Institute Common Terminology Criteria for Adverse Events (CTCAE) version 4.0.

### Statistical analysis

PFS and OS of two specific patients groups were estimated using the Kaplan–Meier method, compared wth the log-rank test. Other comparisons of two specific patient groups were performed using chi-squared test and Fisher’s exact test. Hazard ratios were calculated using a Cox proportional hazard model. Values of *p* < 0.05 were considered significant. Relative dose intensity was the percentage of actual cumulative dose compared with the amount of planned cumulative dose. All statistical analyses were carried out using JMP software (SAS Institute Japan, Tokyo, Japan).

### Ethics approval

All procedures performed in studies involving human participants were in accordance with the ethical standards of the institutional and/or national research committee and with the 1964 Helsinki declaration and its later amendments or comparable ethical standards. This study was approved by the Ethics Committee of Kyushu University Hospital (Approval No. 2019-618).

### Informed consent

Informed consent was not obtained from each patient due to the retrospective nature of the present study. The consent was waived by the Ethics Committee of Kyushu University Hospital.

## Results

### Patients’ characteristics

A total of 28 patients with STS were analyzed. Six and twenty-two patients received eribulin as a first-line treatment and, a second or later-line treatment, respectively. The patients’ characteristics are shown in Table 1. The median age of the patients who received eribulin as a first-line therapy was 76 years (range: 58–82 years), which is significantly older than 62 years (range: 20–76 years) for the patients who received eribulin as a second or later-line therapy. Five patients (83%) were male. Performance status 0 or 1 was observed in three patients (50%). Histology was L-sarcoma, LMS, and others for two patients each. The reason for avoiding doxorubicin therapy was cardiac problems for four patients and advanced age for two.

**Table 1:** Patient’s characteristics.

	Total (n = 28)	First line (n = 6)	Second or later line (n = 22)	*p* value
Median age, years (range)	67 (20–82)	76 (58–82)	62 (20–76)	0.013
**Sex, n (%)**				0.16
Male	14 (50)	5 (83)	9 (32)	
Female	14 (50)	1 (17)	13(68)	
**ECOG PS, n (%)**				0.40
0	2 (7)	1 (17)	1 (5)	
1	13 (46)	2 (33)	11 (50)	
2	11 (39)	2 (33)	9 (41)	
3	2 (7)	1 (17)	1 (5)	
**Histology, n (%)**				0.72
Dedifferentiated liposarcoma	10	2 (33)	8 (29)	
Leiomyosacroma	6	2 (33)	4 (14)	
Others	12	2 (33)	10 (38)	
**Previous chemotherapy, n (%)**				
0	6 (21)	6 (100)	0	
1	9 (32)	0	9 (41)	
> 1	13 (47)	0	13 (59)	
**The reason for avoiding doxorubicin**				
Cardiac comorbidities	4 (14)	4	0	
Old age	2 (7)	2	0	

*ECOG PS* Eastern Cooperative Oncology Group Performance Status.

### Treatment and efficacy

**Figure 1:**
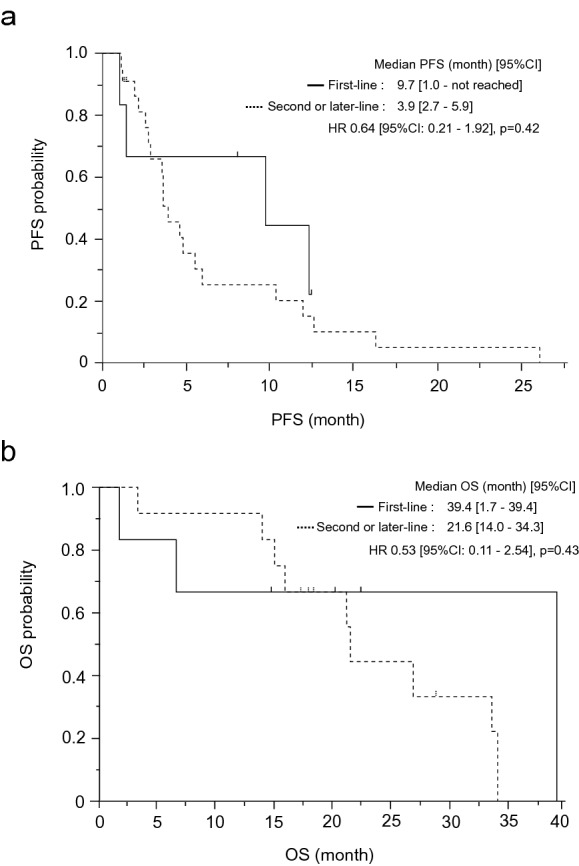
(**a**) Kaplan–Meier plot for progression-free survival (PFS) of patients who received eribulin as a first-line therapy, and second or later-line therapy. 95% CI, confidence interval. HR, hazard ratio. (**b**) Kaplan–Meier plot for overall survival (OS) of patients who received eribulin as a first-line therapy, and second or later-line therapy. 95% CI, confidence interval. HR, hazard ratio.

The median follow-up time was 16 months for patients who received first-line eribulin therapy. At the data cutoff, no patients received eribulin. The reasons for discontinuation of eribulin were disease progression and adverse events (2 fatigue and 1 neuropathy) for three patients each. The median number of cycles of eribulin treatment was 7.5 (range: 2–15). The median relative dose intensity was 93% (range: 49–99%). The reasons for cessation of eribulin treatment were progressive disease and AEs in 50% of the patients each. These AEs were two cases of fatigue and one of neuropathy. Median PFS in the six patients treated with eribulin as a first-line treatment was 9.7 months (95% confidence interval [CI]: 1.0 month–not reached) (Fig. 1a). The PFS rate at 12 weeks was 67%. OS was 39.4 months (95% CI: 1.7–39.4 months) (Fig. 1b). The response rate for the five patients with measurable lesions was 0%, and the disease control rate was 66.7%. On the other hand, the median number of cycles of eribulin treatment for the patients who received second or later-line eribulin therapy was 7.7 (range: 2–22). The median relative dose intensity was 79% (range: 31–100%). The reasons for cessation of eribulin treatment were progressive and surgery in 91% and 9.0% of the patients each. Median PFS was 3.9 months (95% CI: 2.7–5.9 months) (Fig. 1a). The PFS rate at 12 weeks was 65% for twenty patients except for two patients who received surgery. Within twenty-two patients who received eribulin as a second or later-line treatment, twelve patients were initially diagnosed as unresectable or metastatic and received first-line chemotherapy. OS for those patients from first-line chemotherapy 21.6 months (95% CI: 14.0–34.3 months) (Fig. 1b). PFS and OS of the patients who received eribulin as a first-line and, second or later-line therapy were not significantly different: HR 0.64 (95% CI: 0.21–1.92), *p* = 0.42 for PFS, and HR 0.53 (95% CI: 0.11–2.54) *p* = 0.43.

### Safety

All of the patients treated with first-line, and second or later-line eribulin experienced ≥ 1 AE, as shown in Table 2. Grade 4 AEs in patients treated with first-line, and second or later-line eribulin included leukopenia (17% and 14%), neutropenia (33% and 45%) and lymphopenia (17% and 14%). Infection of any grade in patients treated with first-line, and second or later-line eribulin was 50% and 23%. Febrile neutropenia in patients treated with first-line, and second or later-line eribulin was 17% and 14%.

**Table 2:** Summary of Treatment related adverse events.

	First line			Second or later line		
	All Grade, n (%)	Grade 3, n (%)	Grade 4, n (%)	All Grade, n (%)	Grade 3, n (%)	Grade 4, n (%)
Any adverse events	6 (100)	5 (83)	2 (33)	22 (100)	14 (64)	10 (45)
Leukopenia	4 (67)	0	1 (17)	20 (91)	3 (14)	3 (14)
Neutropenia	5 (83)	2 (33)	2 (33)	20 (91)	3 (14)	10 (45)
Lymphopenia	2 (33)	0	1 (17)	15 (68)	3 (14)	3 (14)
Anemia	5 (83)	0	0	20 (91)	2 (9)	0
Thrombocytopenia	3 (50)	1 (17)	0	4 (18)	0	0
Mucositis oral	2 (33)	1 (17)	0	4 (18)	0	0
Sore throat	1 (17)	0	0	2 (9)	0	0
Hiccups	1 (17)	0	0	0	0	0
Constipation	1 (17)	0	0	6 (27)	0	0
Diarrhea	1 (17)	0	0	0	0	0
Appetite loss	3 (50)	0	0	10 (45)	0	0
Fatigue	4 (67)	1 (17)	0	10 (45)	0	0
Alopecia	3 (50)	0	0	3 (14)	0	0
Limb edema	1 (17)	0	0	3 (14)	0	0
Arthralgia	2 (33)	0	0	0	0	0
Myalgia	1 (17)	0	0	1 (5)	0	0
Dizziness	1 (17)	0	0	1 (5)	0	0
Peripheral neuropathy	4 (67)	1 (17)	0	7 (32)	0	0
Rash	2 (33)	0	0	3 (14)	0	0
Skin (Folliculitis)	1 (17)	0	0	0	0	0
Infection	3 (50)	1 (17)	0	5 (23)	1 (5)	0
Febrile neutropenia	1 (17)	1 (17)	0	3 (14)	3 (14)	0
AST elevation	4 (67)	0	0	13 (59)	0	0
ALT elevation	2 (33)	0	0	7 (32)	0	0
ALP elevation	3 (50)	0	0	6 (27)	0	0
Hypokalemia	0	0	0	5 (23)	0	0

*AST* aspartate aminotransferase, *ALT* alanine aminotransferase, *ALP* alkaline phosphatase.

## Discussion

The results of the present study indicate the efficacy of eribulin as a first-line therapy for patients with STS who have contraindications for doxorubicin. No cardiac AEs were observed. However, as many patients with contraindications for doxorubicin are aged, careful safety management of eribulin is necessary.

Here, the efficacy of eribulin is reported for patients with STS previously treated with at least one chemotherapy regimen. PFS was 2.6 months (95% CI: 1.9–2.8) in a phase III trial of previously treated patients^9^. In this trial, 50% of the patients received two previous chemotherapy regimens and 50% received more than two regimens before eribulin. In a phase II trial of eribulin for previously treated patients with STS, PFS was 4.1 months (95% CI: 2.6–5.6)^14^. In this trial, the median number of prior chemotherapy regimens was two (range: 1–7). In the present study, PFS of the patients who received eribulin as a first-line therapy was 9.7 months (95% CI: 1.0–not reached), which is better than that in published reports of previously treated patients. PFS in a phase III trial of doxorubicin as a first-line treatment for advanced or metastatic STS was 4.6 months (95% CI: 2.9–5.6 months)^3^. A previous report showed that eribulin was especially effective for dedifferentiated liposarcoma (DDLS)^15^. The histology of six patients treated in the present trial were DDLS (n = 2), LMS (n = 2), myxofibrosarcoma (n = 1), and unclassified histology (n = 1). PFS of one DDLS and one myxofibrosarcoma patients were censored because of surgery and radiation. PFS of DDLS cases were 12.5 and 12.3 months. PFS of LMS cases were 9.7 and 1.4 months. PFS of myxofibrosarcoma and unclassified histology cases was 8.3 and 1.0 months. Our result was consistent with that of a previous report showing that eribulin had strong potency for DDLS. The response rate for eribulin in patients with measurable lesions was 0%. The response rate for eribulin in previous trials with patients previously treated was 0–4%^7,10^. Our results indicated that the response rate for eribulin was low, even in first-line therapy. PFS of the patients who received eribulin as a second or later-line treatment was 3.9 months (95% CI: 2.7–5.9 months). This result is almost equivalent to the previous report^9^. Although PFS of the patients who received eribulin as first-line and, second or later-line therapy were not significantly different, the efficacy of eribulin as a first-line therapy is considered as meaningful because the population for a first-line eribulin therapy is advanced age and has cardiac problems, and is considered as contraindication for receiving doxorubicin which is a standard first-line therapy.

The reasons for avoiding doxorubicin were cardiac problems for four patients and advanced age for two. Cardiac problems were one case of cardiac function impairment with multiform premature ventricular contraction, one case of hypertrophic cardiomyopathy, and two cases of myocardial perfusion scintigraphy. In the present study, no cardiac AEs were observed.

In addition, 83% of the patients who received eribulin as first-line treatment experienced a grade 3 or higher AE. Febrile neutropenia was observed in 17% of the patients, grade 3/4 neutropenia was observed in 67% of the patients, and grade 3 neuropathy and fatigue were observed in 17% of the patients. Previous clinical trials investigating eribulin for STS reported that 96% of patients experienced a grade 3 or higher AE, including grade 3/4 neutropenia (86.3%) and grade 3 febrile neutropenia (8%), which is comparable with the present trial^10^. However, the rate of any infection in the patients who received eribulin as a first-line treatment was higher than the patients who received eribulin as a second or later-line treatment. Further, 50% of the patients in the present trial withdrew from treatment because of AEs, which is more frequent than the 8% reported in past trials. The AEs related to withdrawal were fatigue and neuropathy. The mean age of the participants in the present trial was 76 years (range: 58–82 years), which was higher than that reported in previous studies (mean: 52 years; range: 28–73 years). This finding suggests that patients with contraindications for doxorubicin tend to be of advanced age, and thus, careful safety management of eribulin is necessary.

The major limitation of the present study is its retrospective design and limited sample size. Further, the approval of eribulin is different between countries. In Japan, eribulin is approved for any kinds of soft tissue sarcoma, but, for example, approved only for liposarcoma in the U.S. Accordingly, the efficacy of eribulin as first-line treatment needs to be more evaluated depending on the approval status of each countries.

In conclusion, the results of the present study demonstrated the safety and efficacy of eribulin for patients inapplicable with contraindications for doxorubicin as a first-line treatment without cardiac AEs. However, careful safety management is necessary because most patients intolerable of doxorubicin are of advanced age.
